# Thoracoscopic enucleation of esophageal leiomyoma in patient with MEN I syndrome

**DOI:** 10.4103/1817-1737.58960

**Published:** 2010

**Authors:** Waleed N. Saleh, Ahmed Bamosa, Hadi Al-Mutairi, Khaled M. Al-Kattan

**Affiliations:** 1 *College of Medicine, Al-Faisal University, Riyadh, Saudi Arabia*; 2 *Division of Thoracic Surgery, Department of Surgery, King Faisal Specialist, Hospital & Research Centre, Riyadh, Saudi Arabia*

**Keywords:** Enucleation, leiomyoma, thoracosocpy

## Abstract

Minimal invasive thoracic surgery is growing rapidly and may become the standard of care for certain diseases. Its benefits over traditional surgery, including reduced morbidities and hospital stay, have been well established in several reports. We herein report a case of midesophageal leiomyoma in a patient with MEN I syndrome successfully enucleated by thoracoscopy highlighting the technical details of the procedure.

Benign tumors of the esophagus constitute less than 10% of all esophageal neoplasm,[1] the commonest of which is leiomyoma which accounts for about two thirds of all benign tumors of the esophagus.[2] On the other hand 10% of gastrointestinal leiomyomas are located in the esophagus and around 90% of cases occur between the ages of 20 and 69 with the male to female ratio of approximately 2:1.[3] The commonest location is the lower esophagus (56%) followed by the middle (33%).[2]

Leiomyomas usually present as single lesion and one-half of the cases are smaller than five cm in greatest dimension. It may remain asymptomatic, but when symptoms arise they are usually in the form of dysphagia, pain and weight loss. The preferred methods for diagnosis are barium swallow and endoscopy. In barium swallow the lesion appears as a smooth rounded filling defect with sharp demarcation[4] and on endoscopy the tumor is freely mobile with intact overlying mucosa, biopsy should be avoided due to the potential risks of bleeding, perforation, and infection. Biopsy may also complicate subsequent enucleation.

We herein present the first case of thoracoscopic excision of esophageal leiomyoma in Saudi Arabia.

## Case Report

**Figure 1:**
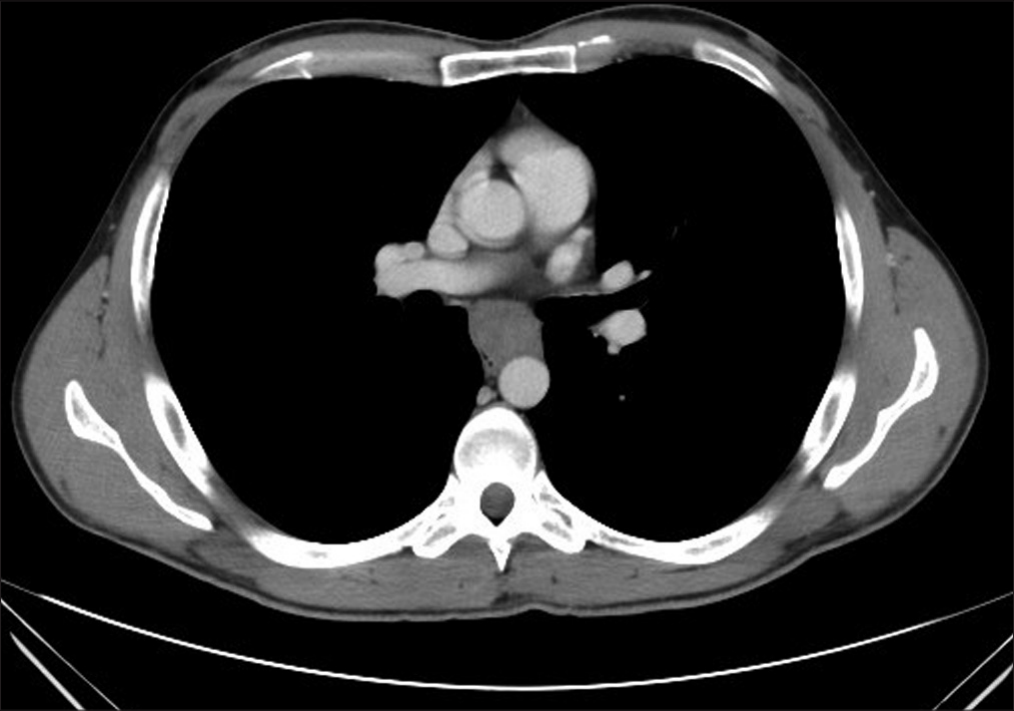
CT scan at the level of the carina showing leiomyoma

A 32-year-old Saudi male patient who is a known case of Multiple Endocrine Neoplasia type 1 (MEN1) status post parathyroidectomy, distal panreatectomy and spleenectomy in 2006 was found, on CT screening, to have extra luminal midesophageal mass about five cm in greatest dimension at the level of the carina, compatible with leiomyoma [Figure 1]. Further review of symptoms revealed mild intermittent dysphagia for solids. Based on these findings the decision was to proceed with thoracoscopic enucleation for which the patient agreed and was consented.

Under general anesthesia utilizing a double lumen tube and in left lateral position, right thoracoscopy was performed after right lung isolation using four ports: 11-mm port is inserted through the sixth intercostal space by blunt dissection at the midaxillary line to provide access for a 10-mm thoracoscope. Three more ports are placed bluntly to provide access for surgery: Five mm port is placed in the posterior axillary line at the fifth intercostal space, 10 mm scope 30 degree is placed in the posterior axillary line at the eighth intercostals space, and a five-mm trocar is placed in the anterior axillary line at the ninth intercostals space for lung retraction.

**Figure 2:**
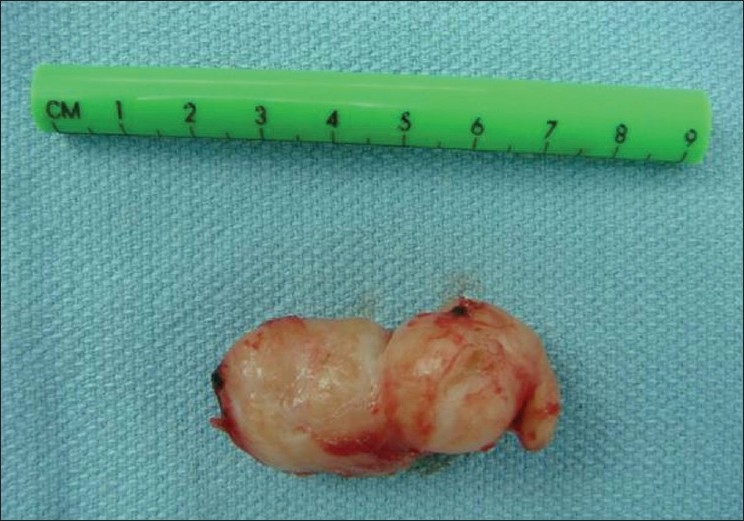
Picture for the leiomyoma after enucleation

The mediastinal pleura over the esophagus were divided to expose the tumor and the adjacent esophagus. About five-cm of the esophagus below the azygus vein was mobilized and the tumor was located. A four-cm myotomy was performed using combination of hook and endoscopic scissor avoiding injury to the mucosa. After that blunt dissection was performed separating the tumor from the mucosa, followed by applying traction suture to the tumor to aid in tumor elevation as well as in the dissection which was done mostly by blunt dissection and occasionally using sharp dissection. After tumor enucleation, the specimen was placed in a retrieval bag introduced through anterior 10-mm trocar and was delivered through this trocar wound [Figure 2].

To ensure that the underlying mucosa is intact, the right thoracic cavity was instilled with normal saline. Size 14F nasogastric tube was inserted proximal to the area of the myotomy and 50-cc of air injected by the anesthetist gently which did not show any leak. The esophageal muscle fibers were approximated using interrupted 2-0 absorbable suture. Size 24F chest tube was introduced through the anterior 10-mm port and directed towards the apex, the trocars were removed under vision, the intercostal muscle fibers were approximated with 2-0 absorbable suture and the skin closed in subcuticular pattern.

The patient was kept nil by mouth till next day when contrast swallow confirmed mucosal integrity, after which he was started on oral fluid sand his drain was removed. On the second postoperative day he was on a soft diet and discharged on the same day. He was to be on soft diet for two weeks. Final histology confirmed the diagnosis of leiomyoma.

## Discussion

Review of literature showed no reports of simultaneous occurrence of MEN syndrome and gastrointestinal leiomyomas. We think this was an incidental finding rather than true association.

Surgical excision of esophageal leiomyoma is advised for symptomatic cases and for asymptomatic cases if the tumor is larger than five cm, if the tumor is increasing in size and if there is mucosal ulceration.[5,6] Esophageal resection is indicated for large lesion (greater than eight cm), if the tumor is densely adherent to the mucosa, or if the there was extensive damage to the mucosa during the dissection that precludes safe repair.[2,7]

Right thoracotomy is the most common procedure used for excision of midesophageal leiomyoma, while distal third lesions are classically approached through left thoracotomy. Upper midline laparotomy may also be used for tumors near the gastro esophageal junction. Muscle fibers are gently separated and the tumor is enucleated by blunt dissection. There is general agreement on approximation of muscle fibers after enucleation to prevent the formation of pseudo diverticulum;[8] however this approximation should be done loosely to avoid decreasing the propulsive activity of the esophageal body. The reported mortality from thoracotomy for leiomyoma excision ranged from 0 to 1.3%;[2] in addition to the well known morbidities of thoracotomy which includes pain, atelectasis, pneumonia and slightly prolonged hospital stay.

This approach has been increasingly used after Everitt reported the first successful thoracoscopic enucleation for esophageal leiomyoma in 1992.[9] A right thoracoscopy is used for proximal two-third lesions and left thoracoscopy for distal-third tumors.

Peroperative endoscopy is an important adjunct to the surgery. It helps in locating the tumor, ensuring complete tumor excision and integrity of the esophageal mucosa. Unfortunately we could not use it in our case.

A review of literature on thoracoscopic excision of leiomyoma found the outcome excellent with majority reporting complete resolution of symptoms. There was no mortality but morbidities included mucosal tear (13.3%), postoperative mucosal bulging (10%) and pleural effusion (3.3%).[8,10] It offers several advantages over thoracotomy including: Less surgical trauma and pain, minimal effect on respiratory mechanics, shorter hospital stay as well as better cosmesis.

Although some surgeons do not routinely perform contrast swallow postoperatively, we prefer to do it in order to ensure mucosal integrity as well as the adequacy of contrast flow at the enucleation site.

## Conclusion

Thoracoscopic enucleation for esophageal leiomyoma is safe and feasible. It should be the procedure of choice for lesions less than five cm since it is less invasive and provides shorter recovery with same functional result to thoracotomy.

## References

[1] Postlethwait RW, Lowe JE, Orringer MB, Zuidema GD (1996). Benign tumors and cysts of the esophagus. Shackelford's surgery of the alimentary tract.

[2] Seremetis MG, Lyons WS, deGuzman VC, Peabody JW (1976). Leiomyomata of the esophagus. An analysis of cases 838 cases. Cancer.

[3] Bonavina L, Segalin A, Rosati R, Pavanello M, Peracchia A (1995). Surgical therapy of esophageal leiomyoma. J Am Coll Surg.

[4] Yang PS, Lee KS, Lee SJ, Kim TS, Choo IW, Shim YM (2001). Esophageal leiomyoma: Radiologic findings in 12 patients. Korean J Radiol.

[5] Zuccaro G, Rice TW, Brandt LJ (1999). Tumors of the esophagus. Clinical practice of gastroenterology.

[6] Fleischer DE, Haddad NG, Castell DO, Richter JE (1999). Neoplasms of the esophagus. The Esophagus.

[7] Hatch GF, Wertheimer-Hatch L, Hatch KF, Davis GB, Blanchard DK, Foster RS (2000). Tumors of the esophagus. World J Surg.

[8] Bonavina L, Segalin A, Rosati R, Pavanello M, Peracchia A (1995). Surgical therapy of esophageal leiomyoma. J Am Coll Surg.

[9] Everitt NJ, Glinatsis M, McMahon MJ (1992). Thoracoscopic enucleation of leiomyoma of the oesophagus. Br J Surg.

[10] Taniguchi E, Kamiike W, Iwase K, Nishida T, Akashi A, Ohashi S (1997). Thoracoscopic enucleation of a large leiomyoma located on the left side of the esophageal wall. Surg Endosc.

